# Dynamic consent framework for low-dose CT scan lung cancer screening: autonomy, privacy, ethical data management

**DOI:** 10.3389/fdgth.2026.1836675

**Published:** 2026-06-10

**Authors:** Jui-Chu Lin, Wesley Wei-Wen Hsiao, Jen-Wei Hu, Chien-Te Fan

**Affiliations:** 1 Graduate Institute of Applied Science and Technology, National Taiwan University of Science and Technology, Taipei, Taiwan, ROC; 2 College of Liberal Arts and Social Sciences, National Taiwan University of Science and Technology, Taipei, Taiwan, ROC; 3 Department of Chemical Engineering, National Taiwan University of Science and Technology, Taipei, Taiwan, ROC; 4 National Center for High-performance Computing, National Institutes of Applied Research, Taipei, Taiwan, ROC; 5 Institute of Law for Science and Technology, National Tsing Hua University, Hsin-Chu, Taiwan, ROC; 6 College of Sustainability, National Tsing Hua University, Hsin-Chu, Taiwan, ROC

**Keywords:** artificial intelligence, blockchain, dynamic consent platform, low-dose computed tomography, lung cancer

## Abstract

**Objectives:**

To develop and implement a blockchain-based dynamic consent framework integrated with artificial intelligence (AI) to support Low-Dose Computed Tomography (LDCT) lung cancer screening and biobank data utilization in Taoyuan, Taiwan.

**Methods:**

We designed a Web 3.0–based dynamic consent platform that enables participants in the Taoyuan Expanded Lung Cancer Screening Program to manage and update their consent preferences digitally. Consent records are secured via blockchain hash registration, while de-identified imaging and biobank data are stored in ISO 27001-compliant infrastructure. The framework incorporates AI-assisted risk assessment and governance mechanisms to ensure compliance with Taiwan's Personal Data Protection Act (PDPA).

**Results:**

This paper presents a conceptual framework and implementation design for a blockchain-based dynamic consent system. The proposed architecture enables real-time consent modification, strengthens data traceability through blockchain hash registration, and improves transparency in data use. The framework is currently being piloted within the Taoyuan Expanded Lung Cancer Screening Program, targeting 15,000 enrolled participants.

**Conclusions:**

A blockchain-enabled dynamic consent system can address legal, ethical, and governance challenges in LDCT-based lung cancer screening programs. This model supports precision health initiatives and provides a scalable pathway for integrating AI and biobank data into public health programs.

## 1 Introduction

Lung cancer has long been recognized as one of the leading causes of cancer-related mortality worldwide, accounting for 1.8 million deaths in both sexes based on the estimated GLOBOCAN 2022 (1). In Taiwan, lung cancer also remains the main cause of cancer mortality at approximately 9,629 deaths reported in 2020, contributing to approximately 19.2% of total cancer deaths (2). The disease's high mortality rate is primarily due to the asymptomatic nature of early-stage lung cancer, leading to late-stage diagnosis. Patients diagnosed at advanced stages have a 5-year relative survival rate of only 3%. In contrast, those diagnosed with localized disease exhibit substantially higher survival rates; therefore, early detection reduces lung cancer mortality and improves survival rates (3). The Low-Dose Computed Tomography (LDCT) scan, which is a type of x-ray that uses a low dose of radiation to create detailed images of the lungs, has emerged as a potential screening method that is currently being piloted on several high-risk groups, including individuals exposed to air pollution or oil smoke, tobacco use, occupational hazards, or who have a personal or family history of lung diseases. Numerous studies have demonstrated that LDCT programs significantly reduce mortality by enabling the early detection of lung cancer in high-risk groups (4, 5). However, these programs have yet to be implemented on a large scale because of the high rates of false positives, limited accessibility due to cost and infrastructure requirements, and inefficiencies in targeting the right populations for screening (6). These limitations underscore the need for complementary tools to enhance the precision and efficiency of LDCT programs. Integrating biobank data, which comprises extensive repositories of genetic, clinical, and environmental factor data, offers a transformative solution for developing more accurate risk prediction models (7). Combining LDCT with biobank-derived datasets enhances the identification of high-risk individuals, optimizes screening resource allocation, and minimizes unnecessary follow-up for low-risk patients.

Biobank data is crucial for LDCT lung cancer screening, enabling researchers to leverage comprehensive genetic, clinical, and environmental information to detect disease early and develop optimal screening strategies. However, using biobank data in research, health policy, and planning is associated with many issues, including data format consistency, data volume, and privacy concerns (8). Addressing these issues is critical to fully realizing the potential of biobanks to improve lung cancer screening outcomes. Artificial intelligence (AI) technology for biobank data offers a potential path forward (8). By leveraging machine learning algorithms, AI can analyze large, complex datasets to identify early-stage disease patterns, leading to tailored screening methods. Recent deep-learning models applied directly to LDCT, such as the end-to-end three-dimensional system reported by Ardila et al., have demonstrated radiologist-level performance for nodule detection on the National Lung Screening Trial dataset, providing concrete evidence that AI can augment LDCT-based screening (9). Integrating AI into screening programs improves accuracy, reduces diagnostic errors, and optimizes resource allocation to ensure timely care for high-risk individuals (7).

Population-scale biobanks linking genetic, clinical, and environmental data have become a foundational resource for risk-stratification research relevant to lung cancer screening, supporting the development of integrated risk models and informing the design of targeted screening programs (7). Building on this evidence base, the present study integrates Taiwanese biobank data with LDCT screening in Taoyuan to enhance lung cancer risk prediction.

However, the use of biobank data and genetic information in LDCT screening raises significant legal and ethical concerns, including data privacy, participant consent, and compliance with national and international data protection regulations (7). To address these challenges, a Dynamic Consent Framework (10) has been incorporated into the LDCT screening program. First articulated by Kaye et al. as a patient-facing interface for twenty-first century research networks, dynamic consent enables participants to review, modify, and withdraw their consent in real time through a digital platform, in contrast to the static, one-time consent typical of traditional research enrolment (10). This framework integrates AI and blockchain technology and empowers participants by providing continuous control over their data and transparency about how their information is used. Unlike traditional consent models, dynamic consent allows individuals to update or withdraw their consent in real time, thereby promoting public participation and trust. For instance, a program using a dynamic consent model was conducted at Tiansheng Hospital in Taoyuan as a part of the city's Expanded Lung Cancer Screening Program. Participants in this model could digitally sign consent forms via the TSBB Precision Health mobile application. The use of standardized protocols and opt-out mechanisms ensures compliance with legal and ethical standards, enabling nationwide scalability. This model's functionalities demonstrate its clinical feasibility and potential for expanding data sources to develop other disease screening programs, contributing to precision health in the future (7, 11). In response to these challenges, the present study describes the design and proposed implementation of a blockchain-based dynamic consent system, hereafter referred to as the Dynamic Consent Framework, within the context of the Taoyuan Expanded Lung Cancer Screening Program.

LDCT program operations begin with participants' registration through the Taoyuan Health Bureau's reservation platform. After eligibility verification, individuals visit one of the contracted hospitals eligible for the LDCT program (contracted hospitals), their identification documents (ID card, health insurance card), and a supplied QR code. The contracted hospitals provide program information, obtain informed consent, and perform the LDCT according to standardized protocols. Results are reported within 6 weeks, and any suspected abnormalities prompt follow-up visits for further evaluation and confirmation.

## 2 Methodology

### 2.1 Assessment of LDCT

LDCT is an effective screening tool for detecting lung cancer and facilitating early intervention and treatment. Since July 1, 2022 (Year 111 in the ROC calendar), the Ministry of Health and Welfare's Health Promotion Administration (HPA) has been promoting the Lung Cancer Early Detection Program (LCEDP), which provides LDCT lung cancer screenings once every two years for high-risk groups, such as heavy smokers or individuals with a family history of lung cancer. To improve Taiwanese public health, Taoyuan City has promoted the “Taoyuan City Expanded Lung Cancer Screening Program” (hereinafter referred to as the Lung Screening Program) since March 1, 2022, subsidizing LDCT screening for high-risk residents over 40 with exposure or family history related to air pollution, tobacco, occupational hazards, or relevant diseases. From March 1 to December 31, 2023 (Year 112 in the ROC calendar), a total of 18,514 participants completed screening and interpretation; 17,273 were negative, 1,241 were positive, and 88 were diagnosed with lung cancer, 76 of which were early-stage (86%). These outcomes demonstrate the program's contribution to reducing lung cancer mortality through early detection. From 2024 to 2025, the program continues to subsidize LDCT screening for eligible applicants and introduces follow-up options for previously positive cases categorised as Category 0, 3, 4A, 4B/4X (hereinafter referred to as “Positive cases”). Participants with previously positive findings may also choose whether to undergo additional value-added screening items, including carotid ultrasound or dual-energy x-ray bone densitometry.

From 2024 to 2025, the Expanded Lung Screening Program continues the implementation framework of the 2023 Lung Screening Program, targeting people with specific lung cancer risk factors, such as air pollution/oil smoke, tobacco use, occupational exposure, and a history of related diseases. In this program, the public fills out basic case information via the Health Bureau of Taoyuan City Government's appointment platform (hereinafter, the “Health Bureau”). Then, the Health Bureau confirms whether they are eligible for the program. Upon confirmation and appointment scheduling, participants are instructed to present a QR code and identification documents (e.g., ID card and health insurance card) to a contracted hospital that meets the eligibility criteria of the program (hereinafter referred to as the contracted hospital), where the contracted hospital will inform the public of the consent form for the examination. The contracted hospitals should send a written results report within six weeks and arrange follow-up visits for any suspected abnormalities, including report review, clinical recommendations, and diagnostic confirmation. In addition, the contracted hospitals should register the results of the LDCT examination on the examination results form and the results of the thoracic specialist's evaluation on the tracking form/evaluation results of the suspected abnormal cases into the Health Promotion Integrated Information System of the Department of Health before the 25th day of the following month. The results of the subsequent tracking or confirmation of the diagnosis should be registered simultaneously. The expected benefits of the Lung Screening Program include enabling early lung cancer detection through LDCT, facilitating timely treatment to reduce incidence and mortality among Taoyuan residents, enhancing public awareness of preventive measures, encouraging regular screening participation, and ultimately lowering healthcare costs and broader social burdens.

The Taoyuan City Lung Screening Program is implemented by (1) the Department of Health, (2) the public who are eligible for the program (hereinafter referred to as the target population), and (3) the contracted hospitals that conduct the screening. The services provided under the program include notification of screening and follow-up test results, subsidies, and incentive payments. In this regard, since the program's implementation is funded by the Taoyuan City Government and carried out by the Department of Health, the Department of Health leads the program's implementation. It is appropriate to clarify the legal relationship between these entities regarding the collection, management, and use of personal information within the overall Program.

Secondly, the types of personal information collected by this program include not only identifying information but also information on habits (e.g., smoking and drinking), family situations, and health and safety records (e.g., occupational diseases, medical reports, treatment and diagnostic records, and test results), of which health records are a special kind of information stipulated in Article 6 of the Personal Data Protection Act (hereinafter referred to as the Personal Data Protection Act). In this case, the lung screening program collects LDCT diagnostic data with the written consent of the patient, which is less limited and less controversial; however, the written consent should comply with the requirements of Article 8, Article 9, and Article 7, Items 1, 2, and 4 of the Personal Data Protection Act.

Thirdly, whether the LDCT diagnostic data collected by the lung screening program, in this case, falls under the scope of the *Regulation on the Management of Human Biological Databases*, and if so, whether this Regulation applies exclusively—since it is considered a special law under the *Personal Information Protection Act* and therefore does not require concurrent application of the general provisions of that Act. In this regard, under Article 3 of the Human Biological Database Regulations, a biological database is one based on a population or a specific group of people for biomedical research and that contains biological samples, natural person data, and other related data and participant information. The biological samples, derivatives, or related data and information are stored non-delinked for subsequent use. This lung screening program aims “to improve the quality of examination, evaluate the effectiveness of the subsidy policy and the follow-up lung cancer screening,” not explicitly “for biomedical research.” Therefore, LDCT diagnostic data collected by the program may be considered a human biological database; however, because of the differing purposes of data collection and the absence of a formally established database by the Taoyuan City Government, it may not meet the qualifications under current regulations. At this stage, it is challenging to consider the applicability of the Human Biological Information Database Act.

In summary, we recommended that the LDCT consent form be revised as follows: According to Article 6(2) of the Personal Information Act, applicable to Article 7(2), consent for any use beyond the specified purpose must be separately expressed and provided in writing form. According to Article 8 of the Personal Information Act, the consent for the original purpose of collection should inform the parties of the following: “I. The name of the public or nonpublic organization. The purpose of the collection. The type of personal information. The period, area, target, and method of utilizing the personal information. The rights and methods that the subject may exercise under the provisions of Article 3. The subject may freely provide personal data and its impact on their rights and interests if they do not provide it.”

Regarding consent for use beyond the specified purposes, Article 6(2) of the Personal Information Act applies to Article 7(2), which requires that individuals be clearly informed of the intended purpose, scope of use, and potential impacts on their rights and interests. The consent shall be a separate expression of meaning and shall be given in writing. In particular, to record the collection purpose and categories of personal data, reference may be made to the “Specific Purposes and Types of Personal Data under the PDPA.”

In addition, the continued multi-purpose use of personal information obtained through the above screening program requires a clearly defined governance process for managing data use beyond the originally specified purposes. However, formally, the screening program has explicitly obtained the public's consent; in substance, the program has established a process for the management of the use of personal information for purposes other than those specified in the original screening program, which includes the following: the requirements for the provision of the information and the application process, the criteria for the approval of the provision of the information, the scope of the provision of the information, and the procedures for the request for the suspension of the use of the information and the deletion of the information. By clearly defining the procedures, the program can meet future demand for more diversified data use while ensuring ethical and legal compliance and maintaining public trust through transparent disclosure.

### 2.2 Study objectives and framework design

**Figure 1:**
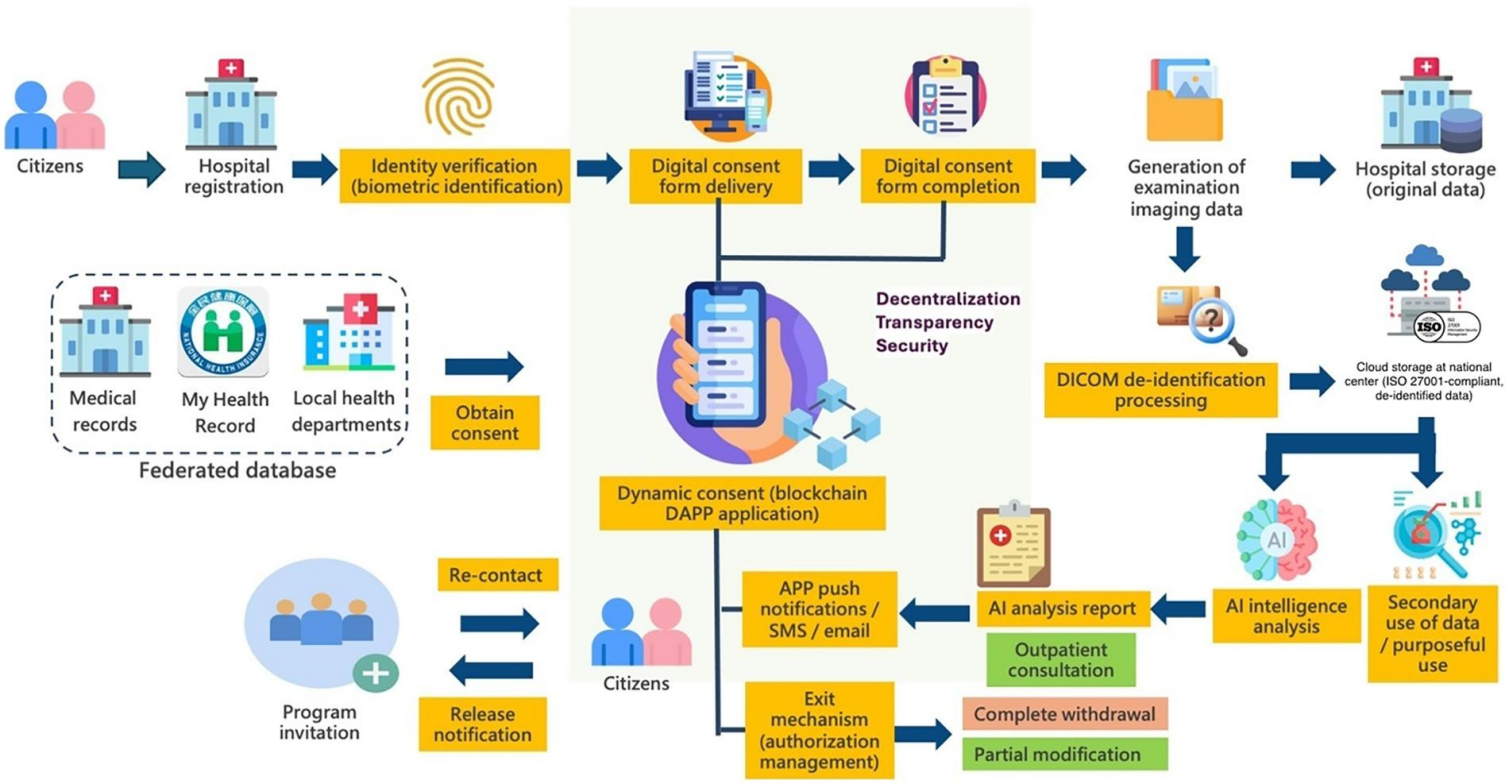
Conceptual architecture of the proposed framework.

This section outlines the objectives and conceptual architecture of the Web 3.0–based Dynamic Consent Framework for the Taoyuan Expanded Lung Cancer Screening Program. The framework is designed to enable participants to manage and update their consent preferences in a secure, transparent, and legally compliant manner. Consent records are secured by registering their cryptographic hashes on a blockchain, thereby supporting tamper-resistant auditability of consent transactions. The conceptual architecture of the proposed framework is illustrated in Figure 1. Note: 17,989 individuals completed LDCT screening in 2023; the dynamic consent pilot targets approximately 15,000 of these participants who are eligible for follow-up and secondary data use under the re-consent procedure. The framework is designed to address re-consent for secondary data use among the approximately 15,000 individuals enrolled in the 2023 Expanded Lung Cancer Screening Program.

The framework is designed to address re-consent for secondary data use among the approximately 15,000 individuals enrolled in the 2023 Expanded Lung Cancer Screening Program. Through a dedicated mobile application (TSBB Precision Health), participants can review, modify, or withdraw their consent preferences at any time, with each transaction recorded immutably on the blockchain. De-identified imaging data is stored at the National Center for High-Performance Computing in compliance with ISO 27001 standards, and participants are provided with opt-out mechanisms and access to relevant program information. With participant authorization, anonymized health data may be utilized for appropriate precision health initiatives.

It should be noted that the framework presented in this paper constitutes a proposed implementation design informed by the operational context of the Taoyuan Expanded Lung Cancer Screening Program. The current phase of this research focuses on system architecture, legal compliance analysis, and consent workflow design. A formal pilot deployment is planned, with prospective evaluation of system usability, consent completion rates, and blockchain transaction integrity. Quantitative implementation outcomes will be reported in subsequent studies.

### 2.3 Innovations in technology resolving legal problems

**Figure 2:**
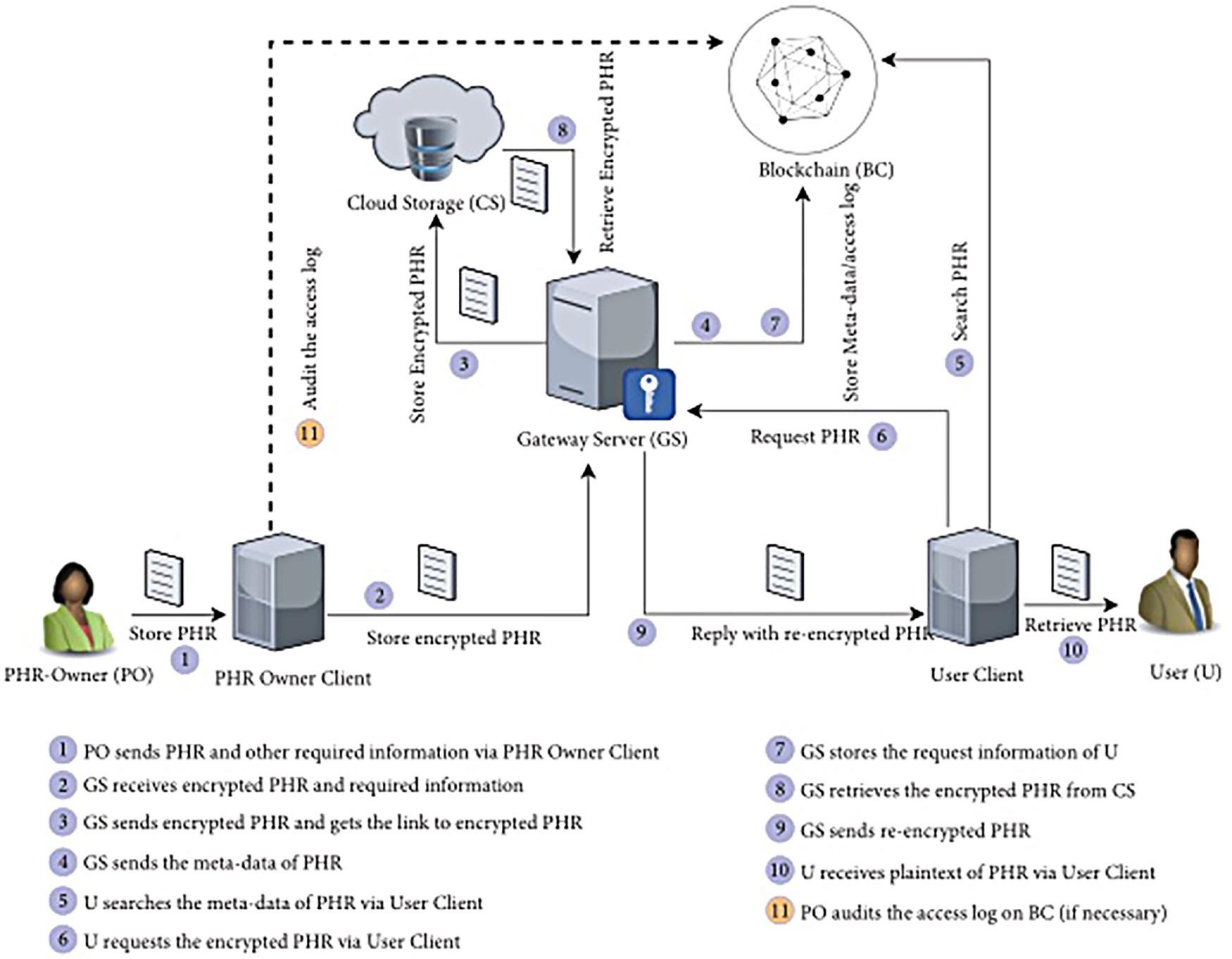
The organization of the system. Reproduced from “The system architecture” by Thein Than Thwin and Sangsuree Vasupongayya, licensed under CC BY.

Technological innovations, particularly blockchain, are essential for addressing legal challenges when applying AI in healthcare settings. In a study by Lin and colleagues (12), large-scale biobanks are becoming increasingly important for facilitating precision medicine research. To address potential legal, ethical, and medical issues, they proposed “hash function” algorithms. Their study highlights the legal and ethical challenges Taiwan Biobank faces in accessing and linking electronic medical records (EMRs) with biobank data, particularly given Taiwan's PDPA. The “hash function” method addresses many countries' challenges: balancing the need for data access in biomedical research with stringent personal data protection laws. Their study offers insights into how legal frameworks such as the PDPA can significantly impact research efforts. They suggest that broad consent models and strong ethical governance could provide a path forward. Their study suggests utilizing algorithms to create anonymized data for cross-transmission and linkage with EMRs. This maintains participant privacy in extensive cohort studies while enabling data sharing for the research (12). Thwin et al. have noted that although traditional cloud-based PHR systems offer benefits such as ubiquitous accessibility and interoperability, they also raise significant privacy concerns. They propose a blockchain-based access control system to protect personal health records (PHRs), recognizing that data retention and tamper-resistance are essential for PHR systems. Blockchain technology addresses this issue by providing tamper-resistant features and supporting the platform with capabilities such as data privacy protection via Proxy Re-Encryption (PRE) and other cryptographic techniques, as well as flexible access control, revocable consent, auditability, and tamper resistance (13). The system is organized as follows (Figure 2).

**Figure 3:**
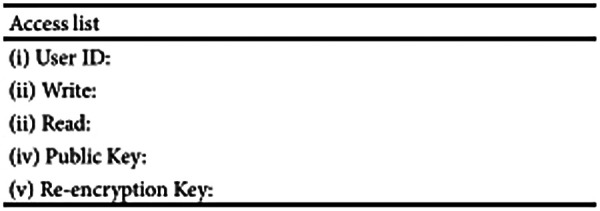
The contents of the access record. Reproduced from “Table 1. The access list and metadata scheme” by Thein Than Thwin and Sangsuree Vasupongayya, licensed under CC BY.

**Figure 4:**
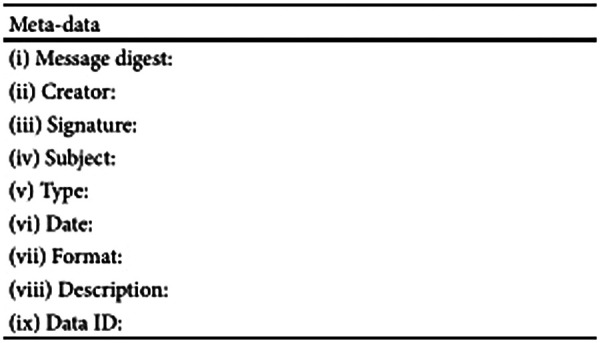
The contents of the meta-data of the stored data. Reproduced from “Table 1. The access list and metadata scheme” by Thein Than Thwin and Sangsuree Vasupongayya, licensed under CC BY.

The given figures depict the access record (Figure 3) and the metadata of the stored data (Figure 4). The owner of the PHR can use the contents of this form to revoke access to any data associated with their PHR, thereby enabling the revocation of consent. However, compared to our approach, their data needs to specify which parts have been de-identified.

Wang et al. proposed a new data-sharing method by creating two blockchains, which they call a double-chain structure; one chain stores the original data, and the other stores transaction data generated by the transactions, thereby separating the storage of original data from transaction data. When combined with the PRE technology, it forms a secure and reliable means of data sharing. However, storing raw data on the blockchain is impractical, and the files must be larger to be processed on the blockchain (14). This measure was developed to address problems with the existing data-sharing model, such as the need for greater transparency in data transactions, the inability to guarantee data security, and inadequate data-tracking methods. There are many sensory devices (e.g., wearable devices, medical devices) that collect EMRs, and the collection of such data, together with artificial intelligence and machine learning, is valuable research material that can be used to improve telemedicine services and promote the development of related healthcare fields. Liu et al. noted that privacy and traceability could not be effectively handled under the traditional centralized data-sharing architecture. Based on decentralized blockchain technology, they propose a traceable and anonymous remote healthcare data storage and sharing scheme with the following features (15): (1) Supporting an anonymous but traceable identity authentication mechanism, which can both protect user's identity privacy by using anonymous identity and reveal the identity of malicious nodes under specific conditions; (2) Designed a batch authentication method to improve authentication efficiency; (3) Considering the need to mitigate medical data sharing, a large amount of data is moved to cloud server storage to reduce storage space, and encrypted storage is utilized to avoid easy viewing of the data. Blockchain technology has been established to store metadata to solve the privacy problem; (4) Data requestors can request data owners to use their data through metadata recorded in the blockchain, and then encrypt the data and send it to the requestors through PRE technology.

In this paper, the data requester makes a single request to the data owner to obtain the content of EMRs for utilization. Still, the system needs a dynamic consent mechanism, a record, and de-identified data storage.

## 3 Proposed system

### 3.1 Workflow

The dynamic consent workflow is structured as follows. Participants enrolled in the Lung Screening Program are invited to register via the TSBB Precision Health application, through which they can digitally review and sign consent forms. Any subsequent modification or withdrawal of consent is recorded in real time, with each transaction hashed and registered on the blockchain to create an immutable audit trail. De-identified imaging data is subsequently transferred to the National Center for High-Performance Computing for secure storage, with access governed by participant-defined authorization settings.

The framework further incorporates an AI-assisted risk assessment module that leverages biobank data, including genetic, clinical, and environmental variables, to support more precise identification of high-risk individuals within the screening program. Recent validated risk-prediction models, such as the Sybil deep-learning system developed by Mikhael et al., demonstrate that future lung cancer risk can be predicted from a single LDCT scan, providing a methodological precedent for the kind of imaging-plus-biobank risk stratification envisaged here (16). This module operates within the consent boundaries established by each participant, ensuring that data utilization remains within legally and ethically defined parameters.

Together, these components form an integrated architecture that addresses participant autonomy, data security, and regulatory compliance under Taiwan's PDPA. The framework is currently in the pre-deployment stage, with a pilot targeting 15,000 enrolled participants planned for subsequent implementation and evaluation.

### 3.2 System architecture ablation analysis

**Figure 5:**
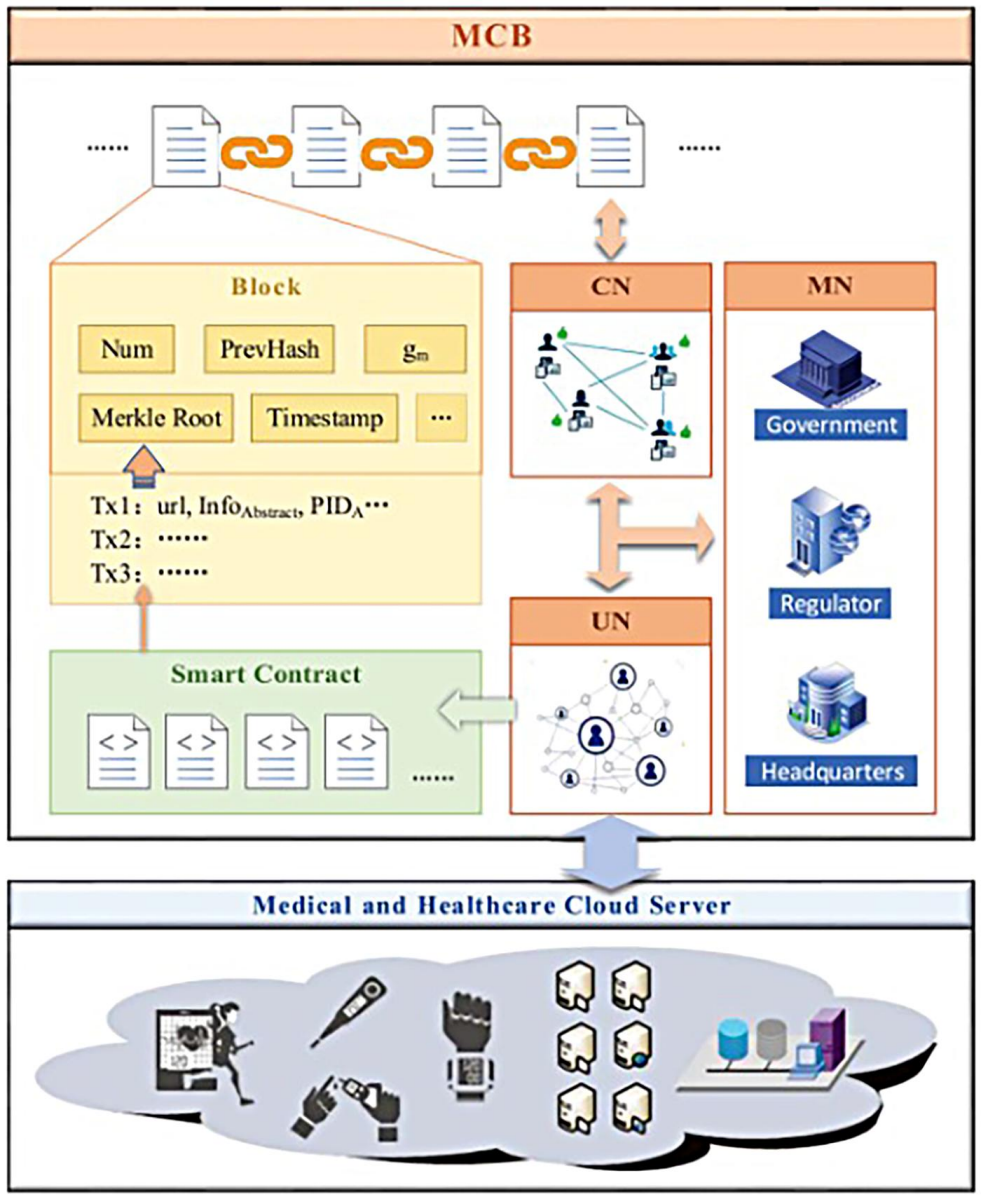
Architecture of the proposed blockchain-based medical and healthcare cloud system.

To further justify the necessity of each architectural layer, a systematic ablation analysis was conducted by establishing a baseline model representing a traditional centralized medical data-sharing system, characterized by paper-based one-time consent, no blockchain integration, and centralized database storage, and evaluating the consequences of removing each layer in turn. The overall architecture of the proposed system is illustrated in Figure 5.

Ablation of the Collection Layer would result in the failure to acquire LDCT images and biobank data in a standardized manner, causing fragmentation of data sources and rendering the AI risk prediction module unable to accurately identify high-risk individuals. Ablation of the Encoding Layer, encompassing de-identification, Proxy Re-Encryption (PRE), and blockchain hash generation, would expose sensitive Personal Health Records and genomic data to transmission in plaintext, creating severe risk of data breaches in direct violation of Taiwan's PDPA.

Ablation of the Storage Layer, which separates ISO 27001-compliant physical infrastructure from blockchain metadata, would either cause network paralysis if raw CT images were stored directly on-chain, or introduce a single-point-of-failure vulnerability if stored in a traditional database alone. Ablation of the Release Layer, comprising the Dynamic Consent application and opt-out mechanisms, would represent a fundamental deprivation of participants' informational autonomy, reverting the system to a broad consent model and violating constitutional protections established under J.Y. Interpretation No. 603 of the Constitutional Court of Taiwan.

Ablation of the Audit Layer, which maintains the immutable blockchain ledger, would eliminate data traceability, rendering the system a “black box” incapable of resolving disputes arising from unauthorized access or AI-assisted clinical interpretations. Ablation of the Accountability Layer, including distributed governance and smart contract access controls, would create ambiguity in responsibility attribution among medical institutions, research facilities, and AI developers, thereby discouraging data sharing among participating entities. Finally, ablation of the Regulatory Layer, which ensures compliance with the PDPA and the Human Biobank Management Act, would render the framework legally undeployable regardless of its technical sophistication.

Taken together, this analysis demonstrates that each layer of the proposed architecture is independently necessary, and that the removal of any single component would compromise either the technical integrity, legal compliance, or ethical standing of the overall framework.

## 4 Discussion

The three key components of the proposed framework, namely dynamic consent, blockchain, and AI-assisted risk assessment, are each independently necessary, as demonstrated by the systematic ablation analysis presented in Section 3.2. Their interdependence is planned to be formally evaluated in future pilot studies through component-specific performance metrics.

### 4.1 Legal and ethical challenges

Incorporating AI and biobank data into LDCT programs holds potential for improving social healthcare but faces legal and ethical challenges (17). The first challenge is that biobank data, which often includes sensitive genetic and health information, must be protected from unauthorized access, complicated by legislative restrictions across nations. For instance, the General Data Protection Regulation in the EU imposes strict privacy requirements that limit international data sharing, and Taiwan's Personal Data Protection Act (PDPA) sets strict guidelines on data handling (12). In particular, interoperability between the proposed framework and GDPR-compliant systems presents notable challenges. Unlike Taiwan's PDPA, the GDPR imposes strict data residency requirements and restricts cross-border transfer of personal data to countries without an adequate protection status. As Taiwan has not yet received an EU adequacy decision, any future international collaboration involving data exchange would require additional safeguards such as Standard Contractual Clauses (SCCs) or binding corporate rules, adding layers of legal and administrative complexity to cross-border deployment of the framework. Although these regulations aim to protect participants' privacy, many barriers remain for researchers, particularly when sharing data, which is crucial for advancing scientific discoveries and conducting large-scale studies. The second challenge relates to the legal problems of AI applications, particularly regarding liability in cases of adverse outcomes (18). A major challenge in implementing medical AI is the lack of clear legal boundaries defining responsibility among physicians, healthcare institutions, and AI developers. The third challenge concerns transparency and accountability, which are further complicated by the “black-box” nature of many AI systems: when multiple actors contribute to the development and deployment of AI in clinical contexts, attributing responsibility for adverse outcomes becomes difficult, particularly where automated decision support shapes clinical interpretation (18, 19). To overcome these challenges, technological and governance frameworks are essential for safely integrating AI into the healthcare (17–19). Blockchain technology provides a secure and transparent system for managing biobank data, ensuring data integrity and compliance with privacy regulations. Blockchain enhances trust and accountability by recording every data transaction on an immutable ledger (7). Moreover, the distributed governance of medical AI systems is crucial for ensuring its safe and effective implementation in diverse healthcare settings because centralized governance models, such as FDA regulations, may not adequately address the varied local contexts where AI is deployed (20, 21).

### 4.2 Polygenic risk scores (PRS)

Polygenic Risk Scores (PRS) estimate an individual's genetic predisposition to disease using large-scale genomic and clinical data from biobanks. PRS enables identification of individuals at high genetic risk for conditions such as coronary artery disease, atrial fibrillation, type 2 diabetes, inflammatory bowel disease, and breast cancer, supporting targeted screening and personalized prevention (22). However, PRS implementation faces major challenges, particularly the limited population diversity in current datasets. Most PRS are derived from Northern European cohorts, which restricts their predictive validity in other ethnic groups, as emphasized by Corpas et al. and Koch et al. (22, 23). This lack of generalizability complicates clinical interpretation and selection of appropriate PRS tools. Recently, Ormondroyd et al. identified consent processes as a central challenge in the generation and use of genomic health data, emphasizing the difficulties of obtaining informed consent for genomic testing and data sharing—particularly in the context of broad consent used in research—as well as broader concerns surrounding data ownership and the commercialization of genetic information (24). Their study proposes developing international regulatory standards, improving patient information, prioritizing public benefit, and exploring public–private collaboration to support responsible precision medicine (24). Suggested strategies for advancing PRS include diversifying biobank datasets, integrating PRS into clinical trials and electronic health records, strengthening education for patients and providers, and addressing legal and ethical considerations (23). These directions are directly relevant to the present framework: the dynamic consent mechanism proposed here is designed to support exactly the kind of granular, time-varying authorisation that responsible PRS deployment requires, including future re-contact for updated risk estimates and participant-controlled withdrawal from secondary uses. A related large-scale initiative is the UK's Our Future Health programme, which aims to enroll five million volunteers to estimate genetic risk for multiple traits and diseases and provide individualized feedback to consenting participants (25).

Recognizing the challenges of managing and developing effective screening mechanisms, our focus on LDCT screening in Taoyuan highlights three priority issues: (1) the protection of autonomy, (2) the notification of incidental findings, and (3) the withdrawal mechanism. Firstly, the consent process must remain within the scope of generalized consent permitted by law, requiring corresponding revisions to the consent form. Secondly, the current plan lacks standardized procedures for managing incidental findings; therefore, establishing a dedicated medical consultation unit is essential to ensure appropriate communication and follow-up. Lastly, as stated in the J.Y. Interpretation No. 603 of the Constitutional Court of Taiwan, “Upholding the dignity of human nature and respecting the free development of the human personality are the core values of the liberal, democratic, and constitutional order. Although the right to privacy is not explicitly enumerated in the Constitution, based on the dignity of human nature, the preservation of individual subjectivity and the integrity of the development of the human personality, and to protect the private sphere of the individual's life from intrusion by others and the autonomous control of personal data, the right to privacy is an indispensable basic right guaranteed by Article 22 of the Constitution (cf. the Court's Interpretation No. 585), which contains the right of the individual to control the information privacy of their data autonomously. It protects people's right to make their own decisions and control the privacy of their data, ensuring the individuals' right to decide whether to disclose their data, to what extent, at what time, in what manner, and to whom. It also protects the people's right to know and control the use of their data and the right to rectify the errors in the data recorded.” The Personal Information Protection Law, the Human Research Law, and the Regulation on the Management of Human Biological Data Banks all provide the right to opt out and to request the cessation or deletion of the collection, processing, or utilization of personal information, which is a manifestation of the protection of personal information autonomy. To ensure the privacy of personal information and to comply with the intent of the Constitutional Court's 111th year Constitutional Judgment No. 13 and J.Y. Interpretation No. 603 of the Constitutional Court of Taiwan, it is advisable to clarify further the planning and design of public opt-out mechanisms.

### 4.3 Evaluation methodology

To ascertain the rigor of the proposed framework, a structured evaluation methodology is planned for the forthcoming pilot deployment. The evaluation is intended to be conducted across four dimensions. First, consent management efficiency is planned to be assessed by measuring consent completion rates, time-to-consent, and rates of consent modification or withdrawal among the 15,000 enrolled participants. Second, system usability is intended to be evaluated using the System Usability Scale (SUS), administered to both participants and healthcare staff involved in program operations. Third, blockchain performance is planned to be assessed through metrics including transaction throughput, confirmation latency, and audit trail integrity under realistic load conditions. Fourth, legal compliance is intended to be verified through periodic audits conducted against the requirements of Taiwan's PDPA and the Human Biobank Management Act, with particular attention to consent record completeness and opt-out mechanism functionality. This evaluation framework is designed to provide empirical evidence supporting the scalability and replicability of the proposed system in broader public health contexts.

## 5 Conclusion

In this study, we proposed a dynamic consent framework incorporating AI and blockchain technologies to address the legal and ethical concerns for the LDCT Lung Cancer Screening Program implemented in Taoyuan by leveraging biobank data to assess the risk of genetic and environmental factors, enabling accurate detection of high-risk individuals and facilitating timely and targeted interventions. As this study presents a conceptual and design-stage framework, formal implementation results are beyond the scope of the current work. Future studies will evaluate the system's real-world performance across four key dimensions: consent management efficiency, system usability, blockchain performance, and legal compliance against Taiwan's PDPA requirements. These evaluations will provide empirical evidence to support broader deployment and policy adoption. This approach can address the legal requirements of Taiwan's PDPA. Furthermore, the framework empowers patients with control over their data, enhancing transparency and fostering participant trust. Blockchain technology further strengthens data security and enables cross-border data sharing, both of which are crucial for advancing international collaborations in medical research. This approach not only improves the accuracy and efficiency of lung cancer screening but also lays the groundwork for future studies, helping optimize current healthcare and advance precision health strategies in Taiwan.

## Data Availability

The raw data supporting the conclusions of this article will be made available by the authors, without undue reservation.
